# Health-care provider compliance with a revised *Plasmodium vivax* radical cure algorithm incorporating tafenoquine and quantitative G6PD testing in the Peruvian Amazon: a prospective observational study

**DOI:** 10.1016/j.lana.2026.101568

**Published:** 2026-07-22

**Authors:** Elisa Vidal-Cardenas, Catharine I. De Freitas-Vidal, Veronica Soto-Calle, Hugo Rodríguez-Ferrucci, Wilma Casanova, Ivan Condori-Lizarraga, Stephan Duparc, Elodie Jambert, Melanie Larson, Heike Huegel, Thy Do, Paula Alejandra Burela, Marcus Lacerda, Alejandro Llanos-Cuentas

**Affiliations:** a MMV Medicines for Malaria Venture, Geneva, Switzerland; b Unidad de Leishmaniasis y Malaria, Instituto de Medicina Tropical Alexander von Humboldt, Universidad Peruana Cayetano Heredia, Lima, Peru; c Universidad Nacional de la Amazonía Peruana, Iquitos, Peru; d Fundação de Medicina Tropical Dr Heitor Vieira Dourado and Fiocruz, Manaus, Brazil

**Keywords:** Plasmodium vivax, Radical cure, Health care provider, Compliance, Tafenoquine, G6PD testing, Malaria treatment guidelines, Implementation science, Peru, Health systems, Provider behaviour, Point-of-care diagnostics, Treatment uptake, Mixed-methods research, Malaria, Malaria elimination, Latin America, Operational research, Real-world evidence

## Abstract

**Background:**

*Plasmodium vivax* relapse remains a key barrier to malaria elimination in Latin America. Historically, radical cure relied on multi-day primaquine regimens, often without prior glucose-6-phosphate dehydrogenase (G6PD) testing. In November 2024, WHO issued a recommendation specific to South America for radical cure with primaquine or single-dose tafenoquine after testing G6PD activity. We assessed the operational feasibility of implementing this revised radical cure algorithm, integrating point-of-care quantitative G6PD testing to guide primaquine or tafenoquine selection, under routine care, measured by health-care provider (HCP) compliance.

**Methods:**

This prospective, observational study in Loreto, Peru (Aug 2022–Dec 2024) evaluated implementation of a revised radical cure algorithm among patients by trained routine HCPs across 14 health facilities. HCP compliance was assessed from the provider (correct algorithm application in ≥80% of patients per HCP role) and patient perspectives (proportion correctly treated per algorithm). Safety endpoints included acute haemolytic anaemia (AHA) and other serious adverse events.

**Findings:**

Among 187 HCPs and 987 patients, HCP compliance was 96.6% (172/178) from the provider perspective and 98.7% (974/987) from the patient perspective, with similar results across urban/periurban and remote/rural facilities. Incorrect treatments (1.3%; 13/987) were mostly due to operational lapses in treatment sequencing and eligibility among G6PD-normal individuals. Ten patients (1.1%; 10/916) developed symptoms suggestive of AHA; one met suspected AHA criteria, unconfirmed. Eight patients (0.8%; 8/986) experienced serious adverse events attributable to severe malaria; all recovered.

**Interpretation:**

HCPs across participating facilities achieved high compliance with the revised radical cure algorithm from both perspectives and consistently across urban/periurban and remote/rural settings. Incorrect treatments were infrequent and no AHA was confirmed. These findings demonstrate that HCP at the participating facilities can successfully implement the revised radical cure algorithm, contributing to the operational evidence base informing its scale-up in Peru and the Americas.

**Funding:**

Unitaid (Grant number 2021-43-VIV).

## Abstract (es)

**Antecedentes:**

La recaída por *Plasmodium vivax* sigue siendo un obstáculo clave para la eliminación de la malaria en América Latina. Históricamente, la cura radical se basaba en regímenes de primaquina de varios días, a menudo sin pruebas previas de glucosa-6-fosfato deshidrogenasa (G6PD). En noviembre de 2024, la OMS emitió una recomendación específica para Sudamérica sobre la cura radical con primaquina o tafenoquina en dosis única, tras realizar pruebas de actividad de G6PD. Evaluamos la viabilidad operativa de implementar este algoritmo revisado de cura radical, integrando pruebas cuantitativas de G6PD en el punto de atención para guiar la selección de primaquina o tafenoquina, en el contexto de la atención rutinaria medido mediante el cumplimiento del personal de salud.

**Métodos:**

Este estudio prospectivo y observacional realizado en Loreto, Perú (agosto de 2022 a diciembre de 2024) evaluó la implementación de un algoritmo revisado de cura radical en pacientes atendidos por personal de salud capacitado en 14 establecimientos de salud. El cumplimiento del algoritmo por parte del personal sanitario se evaluó desde la perspectiva del proveedor (aplicación correcta del algoritmo en ≥80 % de los pacientes por rol del profesional) y del paciente (proporción de pacientes tratados correctamente según el algoritmo). Los criterios de valoración de seguridad incluyeron anemia hemolítica aguda (AHA) y otros eventos adversos graves.

**Resultados:**

Entre 187 profesionales sanitarios y 987 pacientes, el cumplimiento del protocolo fue del 96.6 % (172/178) desde la perspectiva del proveedor y del 98.7 % (974/987) desde la perspectiva del paciente, con resultados similares en establecimientos de salud urbanos/periurbanos y rurales/remotos. Los tratamientos incorrectos (1.3 %; 13/987) se debieron principalmente a fallos operativos en la secuenciación del tratamiento y a la elegibilidad de los pacientes con niveles normales de G6PD. Diez pacientes (1.1 %; 10/916) desarrollaron síntomas sugestivos de AHA; uno cumplió los criterios de sospecha de AHA, sin confirmación. Ocho pacientes (0.8 %; 8/986) experimentaron eventos adversos graves atribuibles a malaria severa; todos se recuperaron.

**Interpretación:**

Los profesionales de la salud de los establecimientos participantes lograron un alto grado de cumplimiento con el algoritmo revisado de cura radical, desde ambas perspectivas y de manera consistente en entornos urbanos/periurbanos y rurales/remotos. Los tratamientos incorrectos fueron poco frecuentes y no se confirmó ningún caso de AHA. Estos hallazgos demuestran que el personal de salud en los establecimientos de salud participantes puede implementar con éxito el algoritmo revisado de cura radical, contribuyendo a la base de evidencia operacional que respalda su ampliación en Perú y las Américas.

**Financiación:**

Unitaid (Número de subvención 2021-43-VIV)

**Research in context**

**Evidence before this study**

Using the search terms “tafenoquine” AND (“feasibility” OR “operational” OR “implementation”) AND “vivax”, we searched PubMed from database inception until Oct 21, 2025, and found 26 studies. After screening the abstracts, we identified four studies reporting the feasibility of tafenoquine implementation following glucose-6 phosphate dehydrogenase (G6PD) activity assessment. Two were qualitative studies, and one was conducted outside the Americas; a single study (the Tafenoquine Rollout STudy—TRuST) from Brazil was therefore directly relevant to the regional context of this work. The study suggested that integration of quantitative G6PD testing and G6PD guided treatment into routine malaria services was feasible. However, important knowledge gaps remained regarding prospective evaluation under routine programmatic conditions, particularly from the perspective of health-care providers (HCPs) responsible for performing G6PD testing, interpreting results, prescribing treatment, and administering therapy according to a revised treatment algorithm in everyday practice.

**Added value of this study**

This study considered HCP compliance with activities related to a revised radical cure algorithm as the main determinant of operational feasibility. Specifically, we assessed HCP compliance with specific assigned activities from both an individual HCP perspective and a patient perspective. Results were reported across urban/periurban and remote/rural health facilities that had varying service capacities. In addition, the prospective nature of this study allowed the identification of safety events among enrolled patients in a contemporaneous manner.

**Implications of all the available evidence**

Complementing the TRuST findings in Brazil, this study provides strong operational evidence that the revised radical cure algorithm can be effectively implemented across diverse health system contexts in Peru. High compliance across 14 participating health facilities demonstrates that HCPs can safely adopt new diagnostic and treatment tools when adequately trained. These results support programme-level implementation and inform regional scale-up across the Americas.

## Introduction

Malaria continues to be a public health challenge in Peru despite considerable progress in recent decades. In 2024, 33,904 cases were confirmed (accounting for 7.5% of the total burden in the Americas) with 85% of these being *Plasmodium vivax* (*P. vivax*) infection.^1^ Transmission is almost exclusively confined to the Amazon rainforest and the northern coastal region, with Loreto in the Amazon bearing the highest endemicity. A large portion of infections stems from relapse of the dormant liver stage of the parasite.^2^ A recent study in Loreto region estimated that relapses accounted for 35–48% of the first PCR-detected *P. vivax* recurrence over 12 months of follow-up.^3^ Achieving national elimination goals requires multiple measures. The integration of effective and well-tolerated *P. vivax* treatment with radical cure—treatment that clears blood- and liver-stages of the parasite—into routine case management plays a critical role.

Since the 1950s, radical cure in Peru has consisted of primaquine as the liver-stage component and standard blood-stage treatment such as chloroquine.^4^ In 2018, following its approval by the United States Food and Drug Administration, tafenoquine became an alternative liver-stage component of radical cure (administered together with chloroquine) for patients aged 16 years and older. Both tafenoquine and the longer multi-day primaquine regimens are recommended by the World Health Organization (WHO) for radical cure of *P. vivax* malaria.^5^ A distinctive feature of tafenoquine is its single-dose administration, offering an advantage over multi-day primaquine. While clinical trials have shown similar efficacy between tafenoquine and primaquine,^6^^,^^7^ the real-world effectiveness of primaquine is often reduced by factors such as poor adherence, variability in cytochrome P450 metabolism, and weight-dependent dosing. In contrast, tafenoquine minimizes adherence-related challenges, may not be affected by cytochrome P450 activity, and uses a standard dose.^8^, ^9^, ^10^, ^11^

Both primaquine and tafenoquine are part of the 8-aminoquinoline drug class that carries a risk of acute haemolytic anaemia (AHA) in individuals with deficient activity of the glucose-6-phosphate dehydrogenase (G6PD) enzyme.^12^ Although the prevalence of G6PD deficiency in Peru is estimated to be less than 2%,^13^ even a single case of drug-induced haemolysis may represent a serious safety concern. Consequently, G6PD screening is essential before administering either primaquine or tafenoquine to mitigate the risk of drug-induced AHA.^5^ In current practice, however, low-dose primaquine continues to be administered without prior G6PD testing.

More affordable and timely quantitative analysis of G6PD activity is now possible through the quantitative G6PD Test (SD Biosensor, Suwon, Republic of Korea), a battery-operated, point-of-care device.^14^ This test measures both G6PD activity and total haemoglobin (Hb), yielding a normalized value (U/g Hb) within 2 min. Given that this approach requires HCPs with varying levels of training to integrate quantitative G6PD testing with radical cure administration, assessing its operational feasibility under real-world conditions is essential.

Two recent real-world implementation studies assessed the operational feasibility of a revised radical cure algorithm integrating quantitative G6PD testing and tafenoquine within national health systems. The Tafenoquine Rollout STudy (TRuST) retrospectively enrolled patients across two Brazilian states and showed that, among patients aged at least 16 years with *P. vivax* mono-infection, 99.7% were appropriately treated or appropriately not treated with tafenoquine in accordance with their G6PD status.^15^ The Assessing Radical Cure Treatment In routine Care (ARCTIC) study was conducted in Thailand with patients from two provinces and showed appropriate use or non-use according to G6PD status in 100% of cases for tafenoquine, 100% for daily 14-day primaquine, and 99.5% for WHO-recommended weekly primaquine.^16^

Building on Peru's political commitment to malaria elimination, the Ministry of Health (MoH) Directorate for the Prevention and Control of Metaxenic Diseases and Zoonoses (hereafter National Malaria Programme) and the Loreto Regional Health Directorate (GERESA Loreto) launched the Malaria Zero Plan in 2018, which later expanded into the National Malaria Elimination Strategic Plan with the goal of eliminating malaria by 2030. Despite progress, the NMP acknowledged WHO's recommendation that achieving elimination would be challenging without tools to improve adherence to radical cure. In consulting with the MoH and drawing on Brazil's TRuST study that was then underway, this study was designed to assess HCP compliance to a revised *P. vivax* radical cure algorithm incorporating quantitative G6PD-guided selection of liver-stage drugs tafenoquine or primaquine into routine practice. The evidence generated is intended to strengthen Peru's national elimination strategy and provide lessons for other Latin American countries.

## Methods

### Study design

**Fig. 1:**
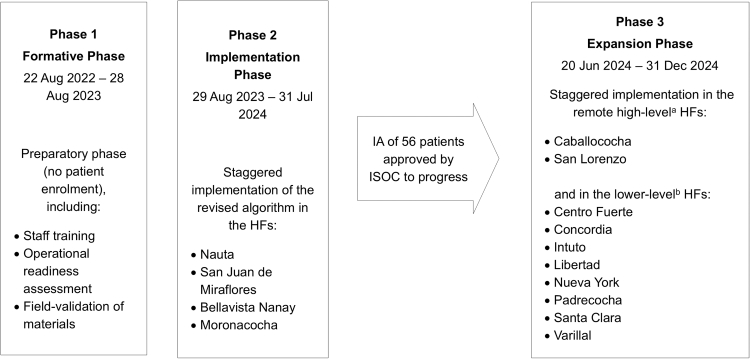
**Phases of implementation of the revised algorithm**. a High-level HFs correspond to first-level care centres with the capacity to manage both outpatient and non-complex inpatient cases. b Lower-level HFs correspond to first-level care facilities without inpatient care. HF: Health Facilities; IA: Interim Analysis; ISOC: Independent Study Oversight Committee.

This descriptive prospective cohort study was conducted across diverse clinical settings in the Loreto region of Peru where GERESA Loreto authorized the use of tafenoquine as part of the standard treatment of uncomplicated *P. vivax* malaria in patients with normal G6PD activity following a revised algorithm (as described below). Implementation of this revised algorithm occurred in three sequential phases (Fig. 1). In Phase 1, future trainers were trained in the use of the revised algorithm and in the evaluation of G6PD measurement. Health facilities were also assessed for their readiness in implementing the revised algorithm within routine services. Phase 2 was carried out in four urban/periurban high-level health facilities. After an interim analysis, an independent oversight committee approved progression to Phase 3, which extended the same approach to two remote/rural high-level facilities and eight lower-level facilities.

The facilities identified in consultation with GERESA Loreto were selected based on the following criteria: (1) caseload of *P. vivax* patients per year (2019–2021 and 2022 until epidemiological week 18); (2) accessibility by road or boat; (3) functional emergency referral system with access to blood transfusion and dialysis; (4) continuous presence of HCPs during the previous year; (5) established process to systematically assess pregnancy status among women of childbearing potential; and (6) alignment with national standard of care.

The study was reviewed and approved by the Universidad Peruana Cayetano Heredia (UPCH) Ethics Committee (104-12-22) and the WHO Ethics Review Committee (ERC.0003718). The study was registered at ClinicalTrials.gov (NCT05361486). A pre-specified study protocol was developed prior to study initiation and is available upon request to the corresponding author.

### Study participants

HCPs were designated as the primary study participants since HCP compliance to the revised algorithm was considered the main determinant of operational feasibility and of the appropriate use of G6PD-guided radical cure with tafenoquine or primaquine. Patients were considered secondary participants because their diagnosis and treatment depended on HCP implementation of the revised algorithm.

All HCPs involved in *P. vivax* case management at the selected facilities were approached by GERESA Loreto through a formal written communication informing them of the temporary mandatory application of the revised algorithm across all presenting non-severe *P. vivax* mono-infected patients.

All patients aged ≥6 months with microscopy-confirmed non-severe *P. vivax* mono-infection who attended the selected facilities underwent G6PD measurement and received treatment according to the revised algorithm and were invited to share data on their current malaria episode, sociodemographic characteristics, and recent malaria history. Pregnant women (contraindicated for both primaquine and tafenoquine) and lactating women (contraindicated for tafenoquine) were also invited, as their cases still allowed assessment of correct case management. Written informed consent for confidential data sharing was obtained from all enrolled participants. All but one invited patient agreed to data sharing. For minors, written informed consent was obtained from a parent or legal guardian.

### Revised case management algorithm

HCP training covered G6PD testing, interpretation of results, treatment decision-making, patient counselling, and the identification and management of AHA (the defined adverse event of special interest for this study). All HCPs involved in malaria management at the selected facilities were required to pass a competency test and, if they failed, received targeted retraining. Patient counselling materials were developed to explain the revised algorithm tools and options, emphasise the importance of follow-up visits, and outline the potential side effects of tafenoquine and primaquine.

**Fig. 2:**
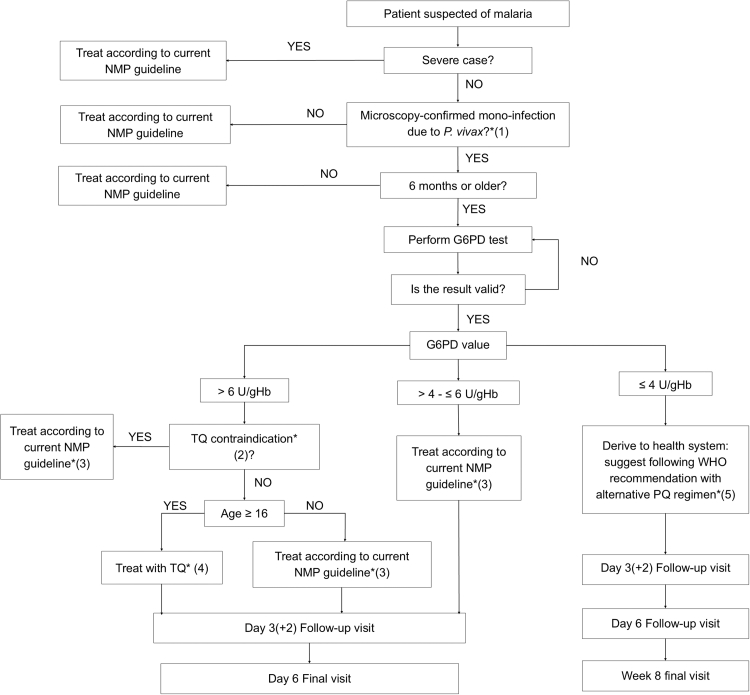
**Revised algorithm for optimised radical cure**. Remarks: a. G6PD test performed on all *P. vivax* malaria non-severe mono-infection cases. b. Blood stage treatment for all cases was CQ per current NMP guideline, which could be given as soon as *P. vivax* mono-infection is confirmed, even before G6PD test result is readily available. c. PQ or TQ is given only after G6PD test result is available. ∗Notes: 1. Ineligibility: patients with decompensated systemic disease, patients currently receiving any antimalarial medications; patients with recurrence of parasitaemia within 28 days after completing treatment. 2. Contraindications for TQ: pregnancy, breastfeeding. If due to travel patient cannot return for the Day 3 and Day 6 follow-up visit, then do not administer TQ. 3. Current NMP guidelines use total dose CQ 25 mg/kg for 3 days and total dose PQ 3.5 mg/kg for 7 days, except pregnant women who only receive CQ weekly; pregnant women are excluded from liver-stage treatment and receive CQ only. 4. Total dose TQ 300 mg (two tablets of 150 mg) single administration. 5. If G6PD ≤ 4 U/gHb: WHO recommends total dose PQ 6 mg/kg weekly for 8 weeks. NMP: National Malaria Programme; G6PD: glucose 6-phosphate dehydrogenase; CQ: Chloroquine; PQ: Primaquine; TQ: Tafenoquine; WHO: World Health Organization; U/gHb: Units per gram of haemoglobin.

The revised radical cure algorithm is shown in Fig. 2. Treatment was given to all patients older than 6 months, but tafenoquine only to patients aged ≥16 years. Patients started treatment on Day 0 with chloroquine in accordance with the National Treatment Guidelines, and tafenoquine 300 mg (single dose administered as two 150 mg tablets) or 7-day primaquine (dosed according to National Treatment Guidelines) was administrated only after obtaining G6PD test results and only if not contraindicated. The first dose was directly observed, after which HCPs were instructed to reinforce the National Treatment Guidelines requirement that patients return to or contact the facility if they experienced any side effects, and to remind those receiving primaquine to complete the full treatment course.

Up to three attempts (by phone or text message) were made to reach patients for the follow-up visit on Day 3, which was scheduled to actively detect early signs or symptoms of AHA that were collected by attending HCPs. This follow-up was in addition to the routine clinical and parasitological evaluation on Day 6, as stipulated in the National Treatment Guidelines. Patients with suspected AHA were referred to the regional hospital for monitoring and management.

Medications included chloroquine (150 mg tablets), tafenoquine (150 mg film-coated tablets; GlaxoSmithKline, Ware, UK), and primaquine (15 mg and 7.5 mg tablets). tafenoquine was supplied directly to selected facilities specifically for the study, whereas primaquine and chloroquine were provided by the NMP from its routine supply stocks using different manufacturers already available at the facility. All facilities were additionally supplied with the STANDARD™ G6PD analysers, test kits, and quality controls.

### Data sources

Data were collected using paper case report forms: one for HCP information (demographic characteristics, educational background, and professional details) and five for patient information (enrolment, current malaria treatment, follow-up, end of treatment, and initial/follow-up visits for every suspected or confirmed AHA). Data were recorded either directly using these case report forms or transcribed from existing facility forms and entered the study database by trained personnel. Sex was self-reported by participants and recorded according to the sex listed on the national identity document, which reflects biological sex at birth. Gender identity was not collected.

### Endpoints

HCP compliance to the revised algorithm was assessed from both the perspective of the HCP and the patient. From the HCP perspective, compliance was defined as correct application of the revised algorithm in at least 80% of the patients managed by that provider. This threshold was established in consultation with Peru's National Malaria Programme as a programmatically relevant benchmark for first-time implementation under routine care. It acknowledges that HCPs perform different roles and were evaluated only on the activities pertinent to their role across up to four sequential activities (i.e., G6PD testing, G6PD results interpretation, treatment prescription, and treatment administration). From the patient perspective, HCP compliance was defined as the proportion of patients who received correct treatment in accordance with the revised algorithm.

Safety data possibly related to AHA were collected prospectively. Patients presenting with new or worsening signs or symptoms of early haemolysis were classified as having suggestive AHA. At this point, haemoglobin was remeasured to assess the severity of anaemia. Patients with a repeat haemoglobin measurement indicating either a decline of >3 g/dL from baseline or an absolute value of ≤7 g/dL at the time of the safety event were further classified as having suspected AHA. Repeat haemoglobin measurements were ideally performed using the same method to ensure comparability. Patients meeting the haemoglobin -based criteria for suspected AHA were promptly referred to the Regional Hospital for admission and further management. When repeat haemoglobin was measured using another method (e.g., HemoCue), only the absolute value was used to guide referral decisions. Final confirmation of AHA was made by the study principal investigator taking into consideration the treating clinician's diagnosis at the Regional Hospital and the admitted patient's clinical history and laboratory test results. Serious adverse events not due to AHA were also recorded at any time during the period in which patients were under routine management by HCPs.

### Statistical analysis

**Fig. 3:**
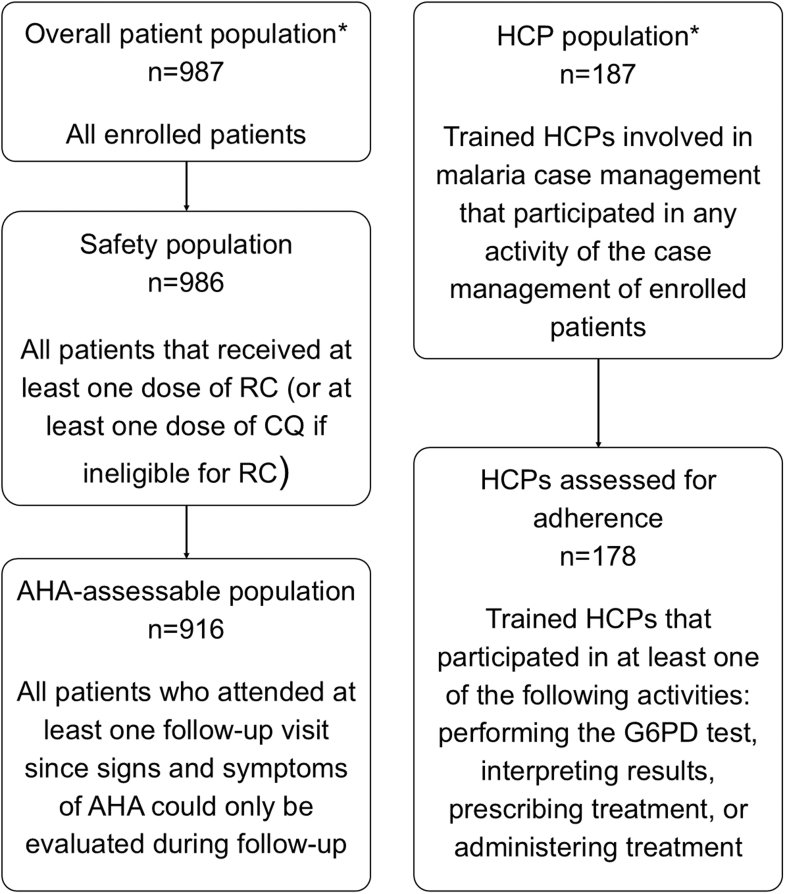
**Study populations**. ∗The study algorithm was applied to all patients attending participating facilities, with all HCPs involved in routine malaria case management trained and expected to apply the study algorithm during the study period. HF: Health Facilities; HCP: Health-care provider; RC: Radical cure; CQ: Chloroquine; AHA: Acute Haemolytic Anaemia; G6PD: Glucose 6-phosphate dehydrogenase.

Analysis populations are noted in Fig. 3. The safety population comprised all patients who received at least one dose of radical cure (or at least one dose of chloroquine if ineligible for radical cure).

Sample size calculations for precision were based on HCPs as the primary unit of analysis. Assuming at least 80% of HCPs would be compliant with the revised algorithm, with a relative error of 15% and a design effect of 1.5, a minimum of 40 HCPs were required. Expecting each HCP to manage approximately 15 *P. vivax* patients, anticipated enrolment was 600 patients. In line with NMP recommendations, however, the study targeted enrolment of 1000 patients.

Descriptive statistics were used to summarise study results, stratified by implementation phase as a proxy for facility-level differences, given that Phase 2 and Phase 3 were conducted at distinct sites.

Missing data were minimal; a ‘missing’ category is reported in Tables 1 and 2 only where applicable, and complete data were available for all primary compliance measures in Tables 3 and 4.

**Table 1:** HCP characteristics, by study phase.

	Phase 2 N = 126	Phase 3 N = 61	Overall N = 187
Sex, n (%)			
Male	68/126 (54.0)	35/61 (57.4)	103/187 (55.1)
Female	58/126 (46.0)	26/61 (42.6)	84/187 (44.9)
Age (years)			
Missing	1	0	1
Mean (SD)	39.1 (9.3)	34.4 (5.4)	37.6 (8.5)
Median (IQR)	37.0 (13)	34.0 (8)	36.0 (11)
Ethnicity			
Missing	1	0	1
Mestizo	123/125 (98.4)	61/61 (100)	184/186 (98.9)
Amazonian native or indigenous	1/125 (0.8)	0	1/186 (0.5)
Caucasian/White	1/125 (0.8)	0	1/186 (0.5)
Professional category, n (%)			
Medical doctor	59/126 (46.8)	13/61 (21.3)	72/187 (38.5)
Obstetrician	0	1/61 (1.6)	1/187 (0.5)
Nurse	8/126 (6.3)	2/61 (3.3)	10/187 (5.3)
Biologist	4/126 (3.2)	2/61 (3.3)	6/187 (3.2)
Laboratory professional	4/126 (3.2)	0	4/187 (2.1)
Nurse technician	5/126 (4.0)	12/61 (19.7)	17/187 (9.1)
Laboratory technician	45/126 (35.7)	31/61 (50.8)	76/187 (40.6)
Pharmacist	1/126 (0.8)	0	1/187 (0.5)
Type of contract, n (%)			
Fixed	37/126 (29.4)	14/61 (23.0)	51/187 (27.3)
Administrative service contract	52/126 (41.3)	32/61 (52.5)	84/187 (44.9)
Rural Service	3/126 (2.4)	4/61 (6.6)	7/187 (3.7)
Other	34/126 (27.0)	11/61 (18.0)	45/187 (24.1)
Experience in managing malaria patients (years)			
Missing	1	0	1
Mean (SD)	8.2 (6.9)	5.0 (3.7)	7.2 (6.2)
Median (IQR)	6.0 (9.3)	4.0 (4.7)	5.1 (7.3)
Training on study received, n (%)			
Yes	125/126 (99.2)	61/61 (100)	186/187 (99.5)
No	1/126 (0.8)	0	1/187 (0.5)

HCP: Health-care provider, SD: Standard deviation; IQR: Interquartile Range.

**Table 2:** Patient characteristics, by study phase.

	Phase 2 N = 634	Phase 3 N = 353	Overall N = 987
Age (years)			
Mean (SD)	33.8 (18.93)	27.2 (18.91)	31.5 (19.18)
Median (IQR)	32.1 (27.4)	22.5 (26.6)	29.2 (28.6)
Ethnicity, n (%)			
Mestizo	574/634 (90.5)	257/353 (72.8)	831/987 (84.2)
Amazonian native or indigenous	60/634 (9.5)	96/353 (27.2)	156/987 (15.8)
Sex, n (%)			
Male	404/634 (63.7)	211/353 (59.8)	615/987 (62.3)
Female	230/634 (36.3)	142/353 (40.2)	372/987 (37.7)
Women of childbearing age (12–49 years), n (%)	140/230 (60.9)	84/142 (59.2)	224/372 (60.2)
Breastfeeding? n (%)^a^			
n	140	84	224
Yes	7/140 (5.0)	9/84 (10.7)	16/224 (7.1)
No	133/140 (95.0)	75/84 (89.3)	208/224 (92.9)
Pregnant? n (%)^a^			
n^b^	140	84	224
Yes^c^	8/140 (5.7)	3/84 (3.6)	11/224 (4.9)
No	110/140 (78.6)	77/84 (91.7)	187/224 (83.5)
Malaria in the last six months, n (%)			
Yes	125/634 (19.7)	99/353 (28.0)	224/987 (22.7)
*P. falciparum*	4/125 (3.2)	9/99 (9.1)	13/224 (5.8)
*P. vivax*	120/125 (96.0)	89/99 (89.9)	209/224 (93.3)
Mixed infection	1/125 (0.8)	0	1/224 (0.4)
Other	0	1/99 (1.0)	1/224 (0.4)
No	507/634 (80.0)	251/353 (71.1)	758/987 (76.8)
Not known	2/634 (0.3)	3/353 (0.8)	5/987 (0.5)
Time since first symptoms from diagnosis of current infection (days)			
Mean (SD)	4.1 (3.21)	3.5 (2.58)	3.9 (3.01)
Median (IQR)	3.0 (3)	3.0 (2)	3.0 (3)
Diagnosis method for current infection, n (%)			
Microscopy	634/634 (100)	353/353 (100)	987/987 (100)
Semi-quantitative G6PD enzyme activity (U/g Hb) groups, n (%)^d^			
Normal ≥6.1 U/g Hb	534/634 (84.2)	290/353 (82.2)	824/987 (83.5)
Intermediate 4.1–6.0 U/g Hb	94/634 (14.8)	60/353 (17.0)	154/987 (15.6)
Deficient ≤4.0 U/g Hb	6/634 (0.9)	3/353 (0.8)	9/987 (0.9)
Haemoglobin level (g/dL)			
Mean (SD)	13.73 (2.275)	13.22 (2.185)	13.55 (2.256)
Median (IQR)	13.80 (3.1)	13.10 (3.2)	13.60 (3.1)
<7 g/dL, n (%)	0	0	0
Liver-stage treatment administered, n (%)			
TQ	379/634 (59.8)	152/353 (43.1)	531/987 (53.8)
7-day PQ	241/634 (38.0)	196/353 (55.5)	437/987 (44.3)
8-weekly PQ	6/634 (0.9)	2/353 (0.6)	8/987 (0.8)
None	8/634 (1.3)	3/353 (0.8)	11/987 (1.1)
Follow-up of patients, n (%)			
Parasite cleared at D7^e^	583/634 (92.0)	259/353 (73.4)	842/987 (85.3)
Parasite not cleared at D7^e^	0	1/353 (0.3)	1/987 (0.1)
Lost to follow up^f^	37/634 (5.8)	84/353 (23.8)	121/987 (12.3)
Withdrawn	0	0	0
Other^g^	14/634 (2.2)	9/353 (2.5)	23/987 (2.3)

SD: Standard deviation; IQR: Interquartile range; G6PD: glucose-6-phosphate dehydrogenase; Hb: Haemoglobin; PQ: Primaquine; D7: Day 7.

a Denominator = number of female patients of childbearing potential (12–49 years).

b Twenty-six women of childbearing age were classified as “not applicable” for pregnancy assessment by certain health-care providers (HCPs) based on individual clinical judgement (e.g., no history of sexual activity, postmenopausal status). This approach was not standardized in the protocol and was not consistently applied across all HCPs.

c In accordance with national treatment guidelines, pregnant women were not eligible for liver-stage treatment (radical cure) and received chloroquine only.

d Among breastfeeding participants (n = 16), 14 were classified as G6PD normal and two as intermediate. Among pregnant participants (n = 11), nine were classified as G6PD normal and two as intermediate.

e Ascertained by light microscopy thick smear reading.

f Defined as patient not returning for parasite clearance assessment on D7 for various reasons (e.g., travel restriction) or unable to be contacted by phone.

g Includes patients who experienced a Serious Adverse Event, those with phone follow-up, and those who did not have a thick smear taken.

**Table 3:** HCP compliance measured from the perspective of the HCP.

	Phase 2 N = 126	Phase 3 N = 61	Overall N = 187
Number of HCPs not involved in any of the following specified activities	5/126 (4.0)	4/61 (6.6)	9/187 (4.8)
Number of patients seen by HCP			
Number of HCPs	121	57	178
Mean (SD)	18.7 (31.2)	16.6 (14.8)	18.0 (27.0)
Median (IQR)	9.0 (17)	13.0 (16)	10.0 (16)
Correctly performing any activity^a^, n/N (%)	120/121 (99.2)	55/57 (96.5)	175/178 (98.3)
Correctly performing G6PD test, n/N (%)	50/50 (100)	29/29 (100)	79/79 (100)
Correctly interpreting G6PD test results and prescribing treatment, n/N (%)	59/61 (96.7)	26/27 (96.3)	85/88 (96.6)
Correctly administering treatment, n/N (%)	17/18 (94.4)	20/22 (90.9)	37/40 (92.5)
Correctly prescribed TQ, n/N (%)	46/48 (95.8)	22/23 (95.7)	68/71 (95.8)
Correctly prescribed PQ, n/N (%)	45/46 (97.8)	23/24 (95.8)	68/70 (97.1)
Correctly performing all activities relevant to the HCP's role, n/N (%)	118/121 (97.5)	54/57 (94.7)	172/178 (96.6)

HCP: Health-care provider; SD: Standard deviation; IQR: Interquartile range; G6PD: glucose-6-phosphate dehydrogenase; TQ: Tafenoquine; PQ: Primaquine.

a Denominators vary by row as each indicator reflects performance among the subset of HCPs in which that specific step of the care pathway (G6PD testing, interpretation, prescription, or administration) was applicable and assessed. Each step was evaluated independently, and may have been performed by the same or different HCPs; therefore, the populations represented in each row are not directly comparable.

**Table 4:** HCP compliance measured from the perspective of the patient.

	Phase 2 N = 634	Phase 3 N = 353	Overall N = 987
Patients receiving any treatment, n (%)	634/634 (100)	353/353 (100)	987/987 (100)
Patients correctly treated with any treatment, n (%)	626/634 (98.7)	348/353 (98.6)	974/987 (98.7)
Patients incorrectly treated with any treatment, n (%)	8/634 (1.3)	5/353 (1.4)	13/987 (1.3)
Patients treated with any radical cure, n (%)	626/634 (98.7)	350/353 (99.2)	976/987 (98.9)
Patients correctly treated with any radical cure, n (%)	618/626 (98.7)	345/350 (98.6)	963/976 (98.7)
Patients incorrectly treated with any radical cure, n (%)	8/626 (1.3)	5/350 (1.4)	13/976 (1.3)
Patients treated with TQ radical cure, n (%)	379/626 (60.5)	152/350 (43.4)	531/976 (54.4)
Patients correctly treated with TQ radical cure, n (%)	374/379 (98.7)	150/152 (98.7)	524/531 (98.7)
Patients incorrectly treated with TQ radical cure, n (%)	5/379 (1.3)	2/152 (1.3)	7/531 (1.3)
Patients treated with PQ (any regimen) radical cure, n (%)	247/626 (39.5)	198/350 (56.6)	445/976 (45.6)
Patients correctly treated with PQ (any regimen) radical cure, n (%)	244/247 (98.8)	195/198 (98.5)	439/445 (98.7)
Patients incorrectly treated with PQ (any regimen) radical cure, n (%)	3/247 (1.2)	3/198 (1.5)	6/445 (1.3)
Patients treated with PQ7 radical cure, n (%)	241/247 (97.6)	196/198 (99.0)	437/445 (98.2)
Patients correctly treated with PQ7 radical cure, n (%)	238/241 (98.8)	193/196 (98.5)	431/437 (98.6)
Patients incorrectly treated with PQ7 radical cure, n (%)	3/241 (1.2)	3/196 (1.5)	6/437 (1.4)
Patients treated with PQ8W radical cure, n (%)	6/247 (2.4)	2/198 (1.0)	8/445 (1.8)
Patients correctly treated with PQ8W radical cure, n (%)	6/6 (100)	2/2 (100)	8/8 (100)
Patients incorrectly treated with PQ8W radical cure, n (%)	0	0	0
Patients treated without radical cure, n (%)	8/634 (1.3)	3/353 (0.8)	11/987 (1.1)
Patients correctly treated without radical cure, n (%)	8/8 (100)	3/3 (100)	11/11 (100)
Patients incorrectly treated without radical cure, n (%)	0	0	0

HCP: Health-care provider; TQ: Tafenoquine; PQ: Primaquine; PQ7: Daily primaquine for seven days; PQ8W: Weekly primaquine for eight weeks.

**Table 5:** Adverse event of special interest (AHA).

	Phase 2 N = 634	Phase 3 N = 352	Overall N = 986
Number of patients who underwent signs/symptoms assessment in at least one FU visit^b^	625/634 (98.6)	291/352 (82.7)	916/986 (92.9)
Number of patients with any new/worsening symptoms	4/625 (0.6)	6/291 (2.1)	10/916 (1.1)
Any new or worsening symptoms reported during any visit, n (%)^a^			
Dark urine (cola coloured)	0	0	0
Yellowing of skin and/or sclera	1/4 (25.0)	0	1/10 (10.0)
Pallor	1/4 (25.0)	1/6 (16.7)	2/10 (20.0)
Fatigue	0	1/6 (16.7)	1/10 (10.0)
Dizziness	0	0	0
Breathlessness or shortness of breath	0	0	0
Back pain	0	3/6 (50.0)	3/10 (30.0)
Rapid heart rate (tachycardia)	0	1/6 (16.7)	1/10 (10.0)
Fever	0	0	0
Nausea and/or vomiting	3/4 (75.0)	0	3/10 (30.0)
Number of patients who had Hb retest			
Not applicable (no Hb retest needed)	621/625 (99.4)	285/291 (97.9)	906/916 (98.9)
Hb retest not performed (retest needed but not done)	2/625 (0.3)	6/291 (2.1)	8/916 (0.9)
Hb retest performed	2/625 (0.3)	0	2 (0.2)
Based on clinical presentation and repeated Hb results^c^, AHA suspected, n (%)			
Yes	1/2 (50.0)	0	1/2 (50.0)
No	1/2 (50.0)	0	1/2 (50.0)

AHA: Acute Haemolytic Anaemia; FU: Follow-up; Hb: Haemoglobin.

a Multiple answers possible.

b Denominators are based on the AHA-assessable population (patients with ≥1 follow-up visit including signs/symptoms assessment; see Fig. 3). Subsequent rows represent subsets of this population.

c One patient had an Hb drop of 4 g/dL, the other patient had an Hb increase of 3.2 g/dL.

Analyses were conducted using SAS V.9.4 (SAS Institute, Cary, North Carolina, USA).

### Role of the funding source

The funder of the study had no role in study design, data collection, data analysis, data interpretation, or writing of the report.

## Results

### Overall HCP population

Characteristics of the overall HCP population (N = 187) are summarised in Table 1. Medical doctors comprised a higher proportion in Phase 2 (46.8%; 59/126) than Phase 3 (21.3%; 13/61), while technicians (both nurse and laboratory) comprised a higher proportion in Phase 3 (70.5%; 43/61) than Phase 2 (39.7%; 50/126). HCPs in Phase 2 also reported a longer mean duration of experience managing malaria patients than those in Phase 3 (8.2 [SD: 6.9] and 5.0 [3.7] years, respectively).

All HCPs received study training, with one exception: a Phase 2 provider who conducted a single G6PD test using the laboratory job aid posted at the facility but left the role before formal training could be provided. This patient was correctly treated.

### Overall patient population

**Fig. 4:**
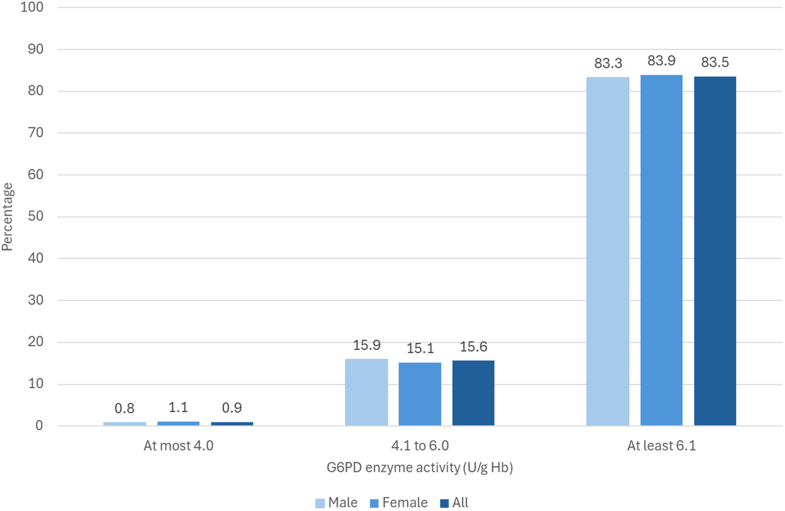
**G6PD enzyme activity categories by sex**. G6PD: glucose 6-phosphate dehydrogenase; U/gHb: Units per gram of haemoglobin.

Characteristics of the overall patient population (N = 987) are summarized in Table 2. Patients in Phase 2 were on average older than those in Phase 3 (33.8 [SD: 18.93] and 27.2 years [SD: 18.91], respectively). G6PD activity categories were similarly distributed between phases, with most (83.5%; 824/987) patients classified as normal and few (0.9%; 9/987) as deficient. Liver-stage treatment differed by phase: most (59.8%; 379/634) patients in Phase 2 received tafenoquine, while most (55.5%; 196/353) patients in Phase 3 received primaquine. All patients received chloroquine as blood-stage treatment. Lost to follow-up was lower in Phase 2 than in Phase 3 (5.8% [37/634] and 23.8% [84/353], respectively). Notably, loss to follow-up was higher in remote/rural higher-level facilities of Phase 3 (39.2%; 69/176) compared with lower-level facilities (8.5%; 15/177). G6PD activity categories was similarly distributed between males and females (Fig. 4).

### Compliance to the revised algorithm

Most HCPs (95.2%; 178/187) participated in at least one revised algorithm-specific activity (i.e., performing the G6PD test, interpreting results, prescribing treatment, or administering treatment). These HCPs attended a median of ten patients each. Overall, HCP compliance measured from the perspective of the HCP (as previously defined) was 96.6% (172/178) for all activities relevant to the HCP's role (Table 3). For the algorithm-specific activity of correctly prescribing treatment, HCP compliance was 95.8% (68/71) for tafenoquine and 97.1% (68/70) for primaquine. Results were similar between phases.

HCP compliance measured from the perspective of the patient (i.e., patients correctly treated) was 98.7% (974/987) for both tafenoquine and primaquine, with similar results between phases (Table 4). No meaningful differences were observed between phases; further stratification by individual facility category or level was not undertaken, as the resulting subgroups would have been too small to yield meaningful or interpretable differences.

Among the seven patients incorrectly treated with tafenoquine, six were G6PD normal and incorrectly treated because chloroquine was started after tafenoquine rather than on the same day (2 patients), age was <16 years of age (2 patients), or breastfeeding was occurring (2 patients). The remaining patient incorrectly treated with tafenoquine was G6PD intermediate and should have received daily primaquine. Among the six patients incorrectly treated with daily primaquine, five were G6PD normal and incorrectly treated because primaquine was started after chloroquine (2 patients); tafenoquine was the correct treatment but was refused (1 patient); tafenoquine was the correct treatment, but primaquine was given due to high blood pressure (1 patient); or the dose was ineffective due to vomiting (1 patient). The remaining patient incorrectly treated with primaquine was G6PD deficient and should have received weekly primaquine rather than daily primaquine.

### Safety

#### Adverse event of special interest (AHA)

Of the 916 patients (92.9%; 916/986) in the AHA-assessable population, ten (1.1%; 10/916, four in Phase 2 and six in Phase 3) reported new or worsening symptoms suggestive of AHA (Table 5). The most frequent symptoms were back pain and nausea/vomiting (three patients each). Among the ten, only two patients (both in Phase 2) had a repeat haemoglobin measured to allow further classification regarding suspected AHA. Based on clinical presentation and the repeat haemoglobin measurement for these two patients, only one patient was considered to have suspected AHA that was ultimately not confirmed as AHA by the study principal investigator. No confirmed cases of AHA were identified. For the remaining eight patients (two in Phase 2, six in Phase 3) without a repeat haemoglobin measurement, HCPs judged patients’ status clinically.

#### Serious adverse events

One patient was excluded from the safety population. This patient died from severe malaria the day after study entry, before receiving primaquine.

From the safety population (N = 986), eight (0.8%) patients experienced a serious adverse event that was not due to AHA. All cases were complications of *P. vivax* malaria, with patients initially enrolled with uncomplicated malaria that later progressed to severe disease after receiving radical cure. These patients were referred to the regional hospital and recovered after receiving appropriate care. All serious adverse events occurred in Phase 2 among G6PD normal patients. The percentage of patients who experienced a serious adverse event was higher with tafenoquine (1.3%; 7/531) than with daily primaquine (0.2%; 1/436); however, all serious adverse events were deemed unrelated to either tafenoquine or primaquine by both the treating physician and the principal investigator.

## Discussion

This study demonstrates that HCPs across 14 facilities in Loreto (Peru) achieved very high compliance to a revised case management algorithm for *P. vivax* malaria integrating quantitative G6PD testing and radical cure with tafenoquine or primaquine. HCP compliance exceeded 95% from both HCP and patient perspectives, reflecting HCPs' ability to correctly apply the algorithm and correctly treat the vast majority of patients. These results underscore the feasibility of introducing new diagnostic and therapeutic tools into routine malaria care when accompanied by structured theoretical and practical training, competency assessments, and job aids. Importantly, compliance remained high across urban/peri-urban and remote/rural facilities, demonstrating that frontline staff could adapt to a new algorithm in diverse service delivery contexts. With adequate training and support, HCPs at the participating facilities successfully applied a complex revised *P. vivax* radical cure algorithm across diverse service delivery contexts, providing Peru's National Malaria Programme with a robust operational evidence base for programme-level adoption.

Observed differences in the study populations between phases largely reflected structural realities of the health system and the patients served. Among the different types of HCPs, Medical Doctors represented a higher percentage in Phase 2 (46.8%) than in Phase 3 (21.3%) while Laboratory Technicians represented a higher percentage in Phase 3 (50.8%) than in Phase 2 (35.7%). This reflected the context of Phase 3 facilities where communities depended more on multi-purpose health staff who undertook several roles within malaria care. Related to the patient population, only 79.6% in Phase 3 attended the Day 3 follow-up visit compared with 97.0% in Phase 2. This reflected greater mobility and transience of rural and border communities that directly impacted treatment (i.e., more primaquine use in Phase 3) since a commitment to a Day 3 follow-up visit was required for tafenoquine eligibility. Notably, the facility that enrolled the most patients in Phase 3 was a remote/rural, high-level facility near the Brazilian border, an area characterised by high cross-border population movement. Furthermore, loss to follow-up was higher in Phase 3, driven mainly by remote/rural higher-level facilities compared with lower-level facilities. As comparison, the TRuST study in Brazil (which was retrospective in study design) reported only 14.2% attendance at the Day 5 visit. Both findings highlight a programmatic vulnerability: the difficulty of sustaining patient engagement beyond drug initiation. Our findings further suggest that, in cross-border regions, loss to follow-up may be shaped by socioeconomic incentives and border trade that drive the frequent pendular movement characteristic of Amazonian frontier areas. These factors should be considered when designing radical cure strategies for border populations. Together, these results reinforce the programmatic value of single-dose tafenoquine in reducing reliance on repeated visits.

Incorrect treatments were infrequent, occurring in 13 patients among 987 enrolled. Only three of these patients were incorrectly treated according to their G6PD status—one G6PD-deficient male patient received a 7-day primaquine regimen, one G6PD-intermediate female patient received tafenoquine, and one breastfeeding patient received tafenoquine although her infant had G6PD unknown status. The remaining 10 patients were incorrectly treated for reasons unrelated to G6PD status. None of the 13 patients, nor the breastfeeding child, experienced any adverse events. The incorrect treatments underscore the importance of reinforcing tafenoquine eligibility criteria and ensuring synchronised initiation of both blood-stage and liver-stage treatments. Although nearly all incorrect treatments occurred in otherwise eligible G6PD-normal patients (which is reassuring from a safety perspective), they nevertheless underscore the operational vigilance required when implementing complex algorithms.

No AHA cases were confirmed. Although ten patients developed suggestive symptoms, only one met the criterion for suspected AHA and was ultimately not confirmed as AHA by the study principal investigator. The limited use of repeat haemoglobin measurements in these ten patients highlights an aspect of clinical evaluation that will need to be addressed in future programmatic settings to ensure timely detection and management of haemolysis. Importantly, this does not call for routine haemoglobin monitoring across all patients. Rather, in the South American context where resources are constrained and G6PD deficiency prevalence is low, targeted haemoglobin assessment triggered by clinical symptoms and signs of AHA represents an appropriate programmatic choice. When clinically indicated, obtaining haemoglobin at baseline to exclude severe anaemia prior to radical cure and repeating it in patients developing symptoms suggestive of AHA is pragmatic and supported by evidence. In settings where dedicated haemoglobin analysers are unavailable, the haemoglobin result from the G6PD testing device already in use offers a readily available adjunct: multi-country clinical validation demonstrates it reliably identifies severe anaemia and outperforms syndromic diagnosis alone.^17^

Beyond haemolysis-related events, eight patients (0.8%; 8/986) experienced a serious adverse event, all attributable to severe malaria and unrelated to tafenoquine or primaquine. However, some patients who were lost to follow-up remained of “unknown” serious adverse events/AHA status, as HCPs were unable to confirm or rule out clinical deterioration in their absence. This underlines the importance of ensuring effective follow-up mechanisms to strengthen pharmacovigilance in settings where return visits are challenging. The occurrence of serious adverse events exclusively in Phase 2 among G6PD-normal patients reflected variability in health system capacity and the limited initial experience of physicians in recognizing severe *P. vivax* malaria early as well as suboptimal management of common complications such as frequent vomiting and dehydration. The severe malaria signs and symptoms outlined in the National Treatment Guidelines and international guidelines proved of limited diagnostic value since cases most often presented with unstable blood pressure that was exacerbated by inadequate assessment of dehydration. Based on lessons learned during Phase 2, Phase 3 trainings emphasized proactive hydration and patient stabilisation before drug administration when needed. This adaptive approach may explain why similar serious adverse events attributable to severe malaria were not reported in Phase 3, even in remote/rural facilities staffed primarily by technicians rather than medical doctors.

Although the study was not designed to estimate G6PD deficiency prevalence, the proportion of G6PD-deficient patients identified (0.9%) is consistent with prior reports from Peru^18^ and with the predominantly Amerindian genetic background of the Peruvian Amazon Basin^19^ and Latin America,^20^ where G6PD deficiency is rare. This contextualises the favourable safety profile observed: the very low proportion of G6PD-deficient patients, combined with the high HCP compliance demonstrated, provides a plausible explanation for the absence of confirmed AHA. This finding aligns with data from GERESA Loreto, which has not reported any case of AHA in the last 20 years. The G6PD deficiency rate observed in Peru contrasts with that reported in the Brazilian Amazon (overall 5.6%; range 4.0–8.3% by state)^20^ and in Brazil's TRuST study (7%),^21^ where a larger proportion of individuals of African ancestry, among whom G6PD deficiency is more common, were enrolled. This contrast underscores the importance of contextualising safety findings within the local epidemiological and genetic background. Notably, the proportion of patients classified as G6PD intermediate (15.6%) was unexpectedly high relative to the very low G6PD deficiency prevalence. As G6PD deficiency is an X-linked trait, populations with very low deficiency prevalence should have correspondingly few intermediates; this disproportionate finding may reflect analytical variability or the use of universal activity thresholds not validated for Peruvian populations,^17^ potentially to overclassification of truly normal individuals as intermediate and therefore tafenoquine-ineligible. This warrants further investigation as the algorithm is scaled.

While the G6PD testing device demonstrates high performance for detecting G6PD deficiency at the 30% activity threshold, its specificity declines at the 70% threshold for intermediate females (98.1% [97.6–98.5]), thereby increasing the likelihood of misclassifying truly normal individuals as intermediates.^17^ This pattern is consistent with the high sensitivity but lower specificity of the assay: in low-prevalence contexts, the positive predictive value decreases,^22^ increasing the likelihood of misclassification. Because the G6PD testing device is a phenotypic assay of enzyme activity, such discrepancies should be interpreted with caution, as activity-based thresholds may vary across populations and do not capture the full spectrum of genetic variants.

The findings on HCP compliance to the revised radical cure algorithm should be interpreted in the context of other recent implementation studies. Whereas Brazil's implementation benefited from a nationwide surveillance platform (SIVEP-Malaria) enabling digital case tracking and hospital linkage, the Peruvian experience demonstrates that high HCP compliance can be achieved under prospective field conditions, including in remote/rural border communities with weaker surveillance infrastructure. Importantly, Peru's study design enabled closer observation of safety monitoring and context-specific operational learnings (e.g., identifying dehydration as a contributor to severe malaria outcomes and adapting training accordingly) illustrating how programmatic refinements can be embedded during implementation and future adoption. Peru updated its malaria treatment guidelines in September 2025 to include tafenoquine and G6PD testing,^23^ representing a direct policy translation of the operational evidence generated by this study. Together, evidence from Brazil and Peru demonstrates that quantitative G6PD testing and tafenoquine can be delivered safely and effectively across diverse health system contexts in the Americas, from highly digitised national programmes to resource-constrained border regions, thereby strengthening the regional evidence base for scaling G6PD-guided radical cure with tafenoquine or primaquine as a cornerstone of malaria elimination.

These findings should be interpreted considering potential sources of bias. The study's enhanced training, monitoring, and oversight, combined with HCP's awareness of being evaluated and the selection of facilities with existing programme capacity, may have led to higher HCP compliance than would be expected in fully routine service delivery. Thus, the results may represent an upper bound of performance at the programme level. Furthermore, the participating facilities were selected to reflect the structure and distribution of malaria services in endemic settings. While not nationally representative, findings from this study may offer relevant insights for facilities with similar caseloads, facility levels, and operational contexts within the Peruvian health system.

This study has limitations that provide important insights for future programmatic implementation. First, although Loreto accounts for most malaria cases in Peru and the 14 participating facilities spanned the full range of facility categories present in the region, findings are most applicable to facilities that regularly manage *P. vivax* malaria. Facilities in lower-endemicity areas, where infrequent case exposure poses distinct operational challenges such as knowledge retention, were not represented; context-specific adaptations will be needed for such settings. Accordingly, our findings are best interpreted as reflecting the experiences of patients and HCPs at participating HFs under routine conditions, rather than those of the wider malaria-affected population in Peru. Second, higher loss to follow-up in remote/rural facilities illustrates how mobility-related barriers could undermine implementation robustness; Strengthening patient tracking systems and adapting follow-up requirements for border and mobile populations will be essential. Third, the study assessed HCP compliance with prescription and administration of the first radical cure dose but did not capture other guideline-required elements such as weight-based dosing accuracy or Directly Observed Therapy. Finally, the small number of suspected AHA cases limits assessment of the Day 3 follow-up visit for early haemolysis detection. Nonetheless, the absence of confirmed AHA and limited repeat haemoglobin measurement in symptomatic patients underscore the need to strengthen pharmacovigilance and clinical monitoring for haemolysis within the Peruvian malaria programme, as increasing numbers of patients receive treatment.

In conclusion, the Peru study provides complementary evidence to the TRuST experience in Brazil, collectively showing that this approach is operationally feasible and well tolerated across diverse health system contexts in the Americas. These convergent findings demonstrate that frontline health services can operationalise this innovation not only in well-resourced national programmes but also in remote, rural and mobile populations. The inclusion of diverse health facility types, from urban/periurban to remote/rural facilities operating under primary-care constraints, and implementation within routine health system enhances the transferability of these findings to other malaria-endemic settings with decentralised health systems and variable access to care. Although differences in epidemiological profiles, health system organisation, and resource availability may require context-specific adaptations, our results are regionally significant and align with evidence from the ARCTIC study in Thailand, underscoring the broader global relevance of quantitative G6PD testing and tafenoquine implementation. As *P. vivax* relapse remains a major obstacle to elimination, the accumulated evidence from Latin America and Asia offers a compelling case for ministries of health and regional partners to accelerate the integration of G6PD-guided radical cure with tafenoquine or primaquine into national strategies, thereby advancing progress towards malaria elimination in the Americas and globally.

## Contributors

EV-C, CD-V, VS-C, SD, EJ, MeL, HH, MaL, and AL-C designed the study and developed/reviewed the protocol. EV-C, CD-V, AB, HH, and TD designed the data collection tools. VS-C, HR, IC, WC, and AL-C coordinated field implementation, data collection, and training of study personnel. IC and WC had direct access to the data and verified the data. CD-V, AB, and IC performed data cleaning and validation. EV-C, CD-V, AB, and TD oversaw data analysis and interpretation. TD wrote the first draft of the report with input from EV-C, CD-V, and HH. EV-C, CD-V, AB, SD, EJ, MeL, HH, and TD provided overall study supervision. EV-C, EJ, MeL, and SD were involved in funding acquisition. All authors had full access to the data, critically reviewed the paper, approved its submission, and accept responsibility for its contents.

## Data sharing statement

De-identified participant data are available on reasonable request and with completion of a signed data access agreement from https://www.mmv.org/about-us/contact-us, referencing this publication. Data will be available for at least 5 years from publication of this study.

## Declaration of interests

Stephan Duparc was a full-time MMV employee during the study and transitioned to a consulting role during manuscript preparation. Elodie Jambert, Melanie Larson, and Thy Do were full-time MMV employees during the study and manuscript preparation. Elisa Vidal-Cárdenas, Catharine de Freitas-Vidal, Paula Alejandra Burela, and Heike Huegel served as MMV consultants during the same period; Elisa Vidal-Cárdenas remains engaged as an MMV consultant. MMV sponsored this study, funded by Unitaid (Grant 2021-43-VIV); payments corresponded to normal remuneration with no personal financial interest in study outcomes. Verónica Soto-Calle, Hugo Rodríguez-Ferrucci, Wilma Casanova, Ivan Condori-Lizarraga, and Alejandro Llanos-Cuentas were contracted by UPCH, the implementing institution, which received Unitaid funding via MMV; no payments were made directly to these authors. Hugo Rodríguez-Ferrucci holds a remunerated position on the Equipo Técnico de Metaxénicas, DIRESA Loreto, with no financial interest in this study. Wilma Casanova holds an unpaid role as Dean of the Consejo Regional VI, Colegio de Enfermeros del Perú. All authors declare no other competing interests.
